# Case Report: Fatal Multiorgan Failure and Heterochronous Pneumonitis Following Pembrolizumab Treatment in a Patient With Large-Cell Neuroendocrine Carcinoma of Lung

**DOI:** 10.3389/fphar.2020.569466

**Published:** 2021-01-29

**Authors:** Xiaohong Xie, Fei Wang, Yinyin Qin, Xinqing Lin, Zhanhong Xie, Ming Liu, Ming Ouyang, Bihui Luo, Yingying Gu, Shiyue Li, Dejian Gu, Rongrong Chen, Chengzhi Zhou

**Affiliations:** ^1^ State Key Laboratory of Respiratory Disease, Department of Pulmonary and Critical Care Medicine, Guangzhou Institute of Respiratory Health, National Clinical Research Center for Respiratory Disease, The First Affiliated Hospital of Guangzhou Medical University, Guangzhou, China; ^2^ First Clinical College, Guangzhou Medical University, Guangzhou, China; ^3^ Department of Cardiovascular Medicine, The First Affiliated Hospital of Guangzhou Medical University, Guangzhou, China; ^4^ State Key Laboratory of Respiratory Disease, Department of Respiratory Pathology, Guangzhou Institute of Respiratory Health, National Clinical Research Center for Respiratory Disease, The First Affiliated Hospital of Guangzhou Medical University, Guangzhou, China; ^5^ Geneplus‐Beijing, Beijing, China

**Keywords:** immune-related adverse events, myasthenia gravis, myocarditis, pembrolizumab, pneumonitis

## Abstract

Immune checkpoint inhibitors have radically changed the landscape of antitumor therapies in several malignancies. Despite the long-term efficacy, severe immune-related adverse events (irAEs) were not uncommon. However, fatal simultaneous multiorgan failure was rare. Here, we described a patient who developed multiorgan failure, including fulminant myocarditis, myasthenia gravis crisis, hepatic dysfunction, and delayed pneumonitis after pembrolizumab therapy for lung large-cell neuroendocrine carcinoma. After failure of high-dose steroid treatment, implantation of cardiac pacemaker combined with high-dose steroids successfully controlled myocarditis caused by immune checkpoint inhibitors (ICIs). Delayed pneumonitis occurred unexpectedly, and it was treated successfully with steroids. With wild adoption of ICIs in clinical practice, investigations for predictive markers of irAEs are warranted, and more successful treatment strategies are worth sharing.

## Introduction

Large-cell neuroendocrine carcinomas (LCNECs) account for approximately 3% of all lung cancers and are high-grade neuroendocrine carcinoma (Lantuejoul et al., 2020). LCNECs are very aggressive, with 5-year overall survival rates below 15–25% (Travis et al., 2015). Surgical removal has to be considered each time if it is possible and perioperative chemotherapy has been suggested to be beneficial to patients with resected LCNEC (Abedallaa et al., 2012). For advanced stages, there is no standard of treatment for LCNEC, which can be treated by either small-cell lung carcinoma- (SCLC-) type chemotherapy (platinum-etoposide based) or by non-small-cell lung carcinoma- (NSCLC-) type chemotherapy regimens (gemcitabine/taxane/pemetrexed combined with platinum) (Masters et al., 2015; Lantuejoul et al., 2020). Targeted therapy for LCNEC patients is still being explored (Derks et al., 2018); and research has also pointed out that LCNEC patients can benefit from immune checkpoint inhibitors (ICIs) (Kim et al., 2018; Zhuo et al., 2019). Clinical trials have recently shown therapeutic benefits of monoclonal antibodies against immune checkpoints, including cytotoxic T-lymphocyte-associated antigen 4 (CTLA-4), programmed cell death protein 1 (PD-1) and its ligand PD-L1, or combined therapy of ICIs for neuroendocrine tumor (Pardoll, 2012; Horn et al., 2018; Chung et al., 2019).

With wide application, system-wide adverse events (AEs) associated with ICIs have been noted. The most common AEs were dermatologic, hepatic, and endocrine toxicities (Komaki et al., 2018). In contrast, cardiovascular toxicities, including myocarditis, a potentially fatal clinical disease, were among the rare organ toxicities of ICI therapy (Matson et al., 2018; Nasr et al., 2018). The incidence of myocarditis is ∼0.06% in nivolumab treatment and relatively higher with combination blockade (0.27%) of ipilimumab (Johnson et al., 2016). To our knowledge, fatal simultaneous multiorgan failure has rarely been reported (Charles et al., 2019; Hyun et al., 2019). Here, we reported a case where cardiac pacemaker and high-dose steroids successfully controlled the simultaneous fulminant myocarditis, myasthenia gravis crisis, and hepatic dysfunction, and delayed pneumonitis in a patient with lung LCNEC caused by pembrolizumab plus chemotherapy.

## Case Report

**FIGURE 1:**
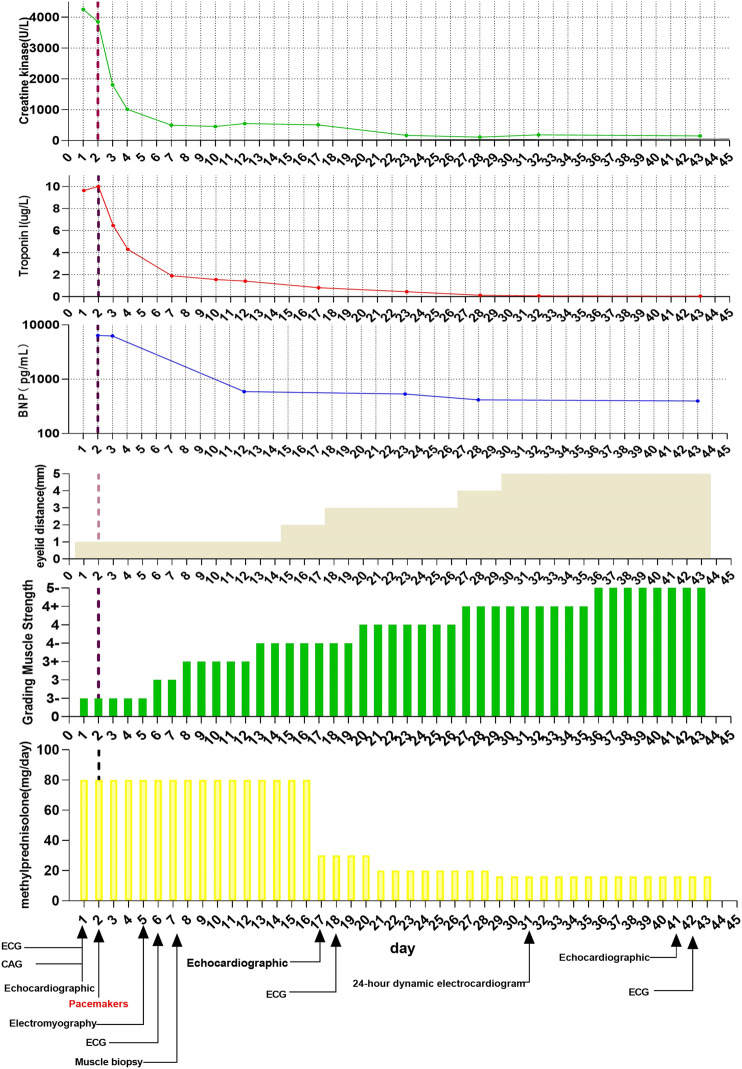
Dynamic changes in patient treatment and biochemical indicators.

**FIGURE 2:**
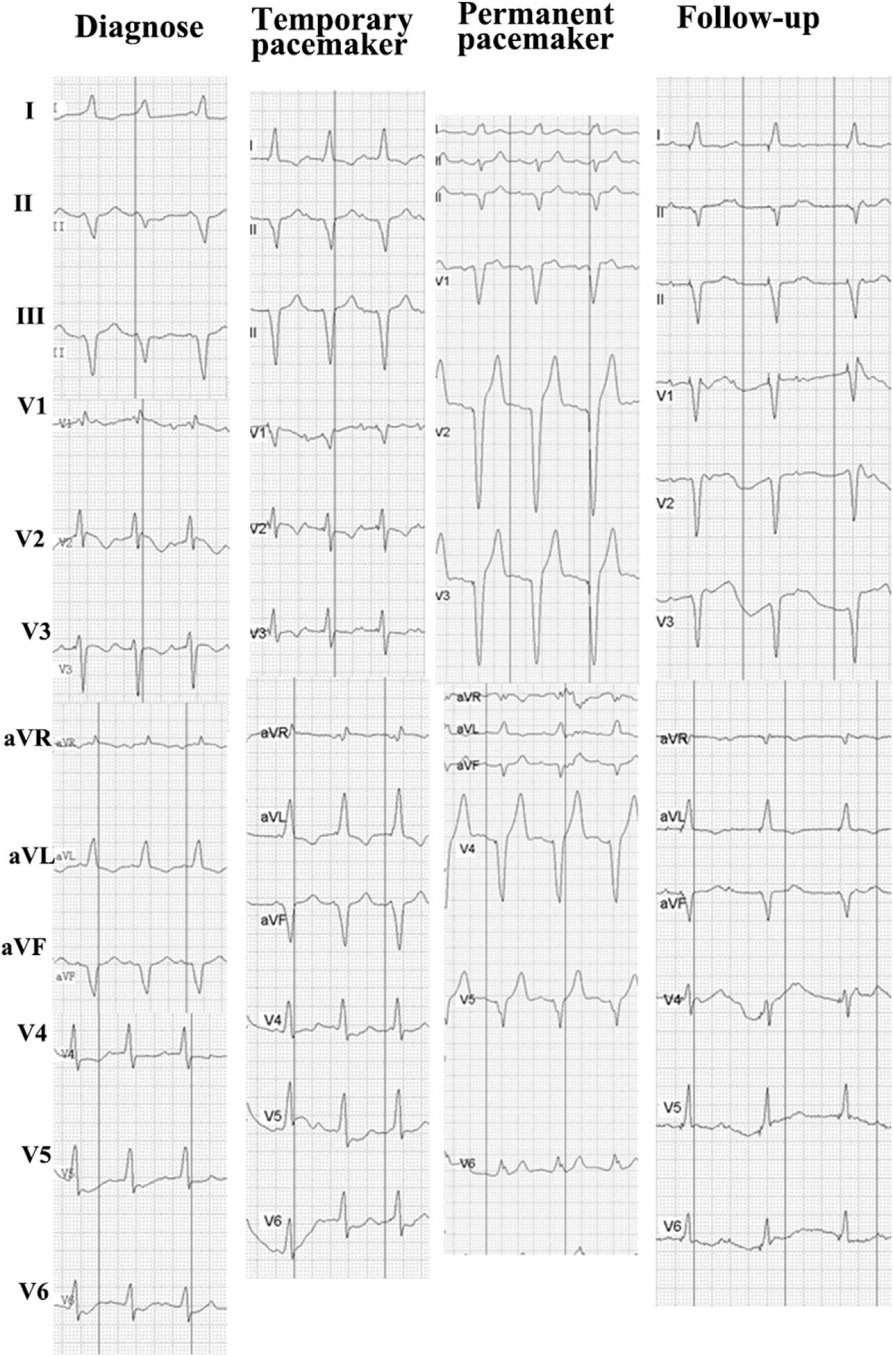
ECG siagnosis, which showed a complete left bundle branch block and monitoring ECG of treatment.

A 67-year-old Chinese male patient was treated in our hospital for lung LCNEC (T4N2M1c, bone metastasis). He had no history or risk factors of cardiovascular diseases. As no EGFR mutations or ALK fusions were detected, the patient received first-line therapy of combined pembrolizumab (200 mg), pemetrexed (900 mg), and carboplatin (400 mg) in May 2019. Two weeks after the first therapy of pembrolizumab, the patient presented with dyspnea on exertion, ptosis (1 cm), blurred vision, and quadriparesis. Laboratory tests revealed remarkable elevation: creatine phosphokinase (CK) was 4256.0 U/L (normal: 38–174 U/L); creatine kinase MB isoenzyme (CK-MB) 109.0 U/L (normal: 0–24 U/L); brain natriuretic peptide (BNP) 6,390.00 pg/ml (normal: 0–99 pg/ml); troponin I 9.64 ng/ml (normal <0.014 ng/ml); myohemoglobin 1,943.7 ng/ml (normal: 20–80 ng/ml). Moreover, liver function test results also showed elevation of aspartate aminotransferase (AST), 661 U/L (normal: 15–40 U/L), and alanine aminotransferase (ALT), 212 U/L (normal: 9–50 U/L) (Figure 1). The electrocardiogram (ECG) showed complete left heart block (Figure 2). The echocardiogram demonstrated a subnormal left ventricular ejection fraction (EF, 50%), and no abnormalities were found by coronary angiography (Supplementary Figure S1). Hence, the patient was considered to have simultaneous multiorgan failure, including fulminant myocarditis, myasthenia gravis crisis, hepatic dysfunction, and ptosis, caused by ICIs.

**FIGURE 3:**
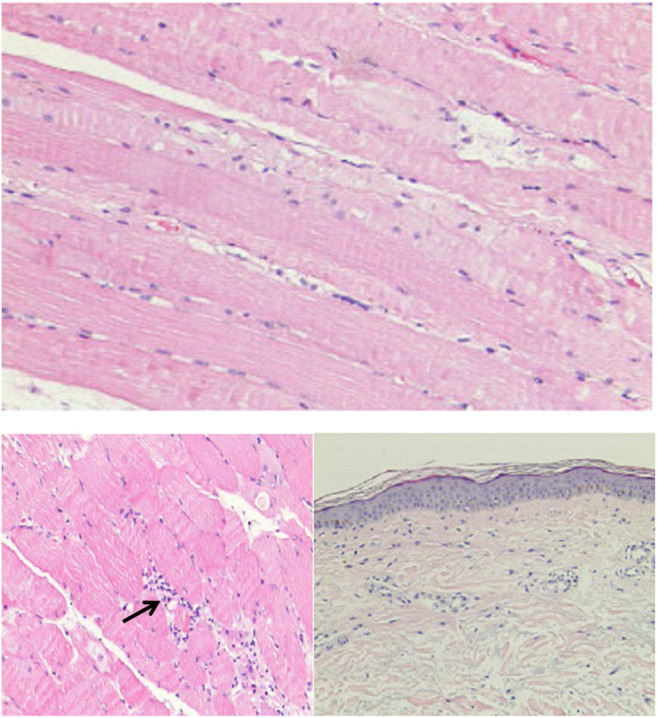
The histological examination of muscle tissue biopsy specimen. Black arrow showed focal lymphocyte infiltration.

Although cardiac magnetic resonance imaging and endomyocardial biopsy could not be performed due to rapid deterioration of symptoms, myocarditis was strongly suspected owing to the presence of typical clinical and laboratory features. High-dose intravenous steroid therapy (1-day methylprednisolone 1 mg/kg followed by 80 mg/day pulse therapy for 2 days) was administered. After therapy with high-dose intravenous steroid, arrhythmia did not improve significantly. Then, a temporary pacemaker was implanted and steroid therapy was continued (Figure 2). With the continuous treatment, the CK, BNP, and troponin I decreased gradually and returned to normal (Figure 1). Considering the patient’s clinical situation, we replaced the temporary pacemaker with a permanent pacemaker on the eighth day of implantation (Figure 2). Muscle biopsy indicated partial myofibril cavity degeneration, myolysis, and most of the striated interstitium showed focal lymphocyte infiltration, which was consistent with drug-induced myopathy, and it supported the original diagnosis (Figure 3). The patient was discharged from the hospital on the 43rd day for significant improvement in symptoms of heart failure (Supplementary Table S1).

**FIGURE 4:**
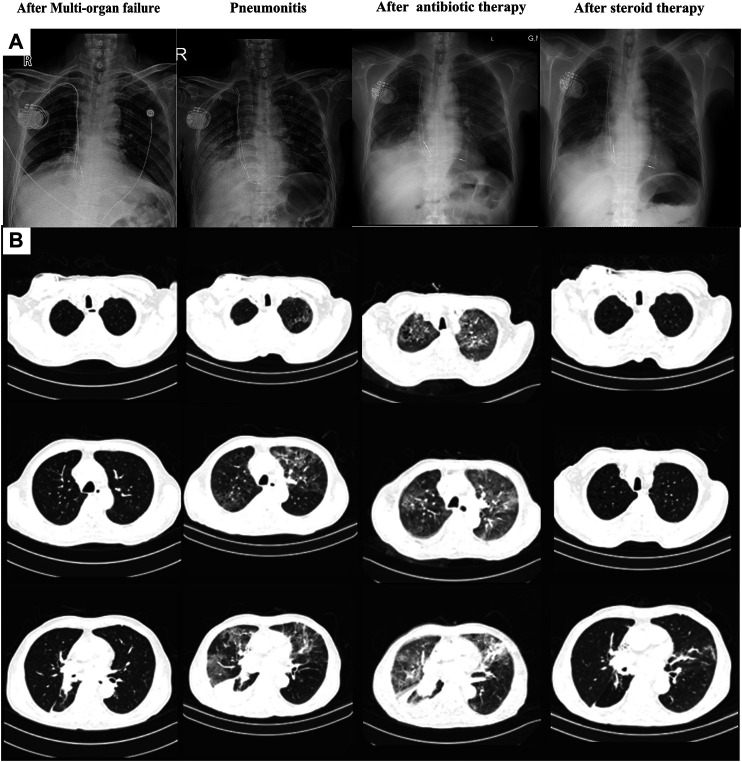
Chest X-ray **(A)** and computed tomography **(B)** of the process of pneumonitis finding and treatment.

Unfortunately, the patient presented to our hospital with dyspnea on exertion after 2 weeks. His body temperature was 36.5°C, blood pressure was 140/85 mmHg, heart rate was 110 bpm, and percutaneous oxygen saturation was 92%. Coarse breathing sounds with wheezing were noted on auscultation of the bilateral lung fields. Procalcitonin (PCT) was 0.20 ng/ml, and no abnormalities were found in the other tests, including cardiac markers and liver function test. Chest X-ray imaging and computed tomography (CT) showed percolate increasing of lung (Figure 4). The patient was treated with antibiotics (ganciclovir and cefmetazole) for 7 days; however, his dyspnea did not improve and chest X-ray imaging and CT showed increasing of percolate (Figure 4). Delayed immune-related pneumonitis was considered and steroid (methylprednisolone, 40 mg per day) and antibiotic (ganciclovir/cefmetazole) therapies were adopted. His symptoms were significantly improved after 3 days of methylprednisolone therapy (Figure 4). On the 3-month follow-up, the symptoms of this patient did not recur.

## Discussion

With the wide application of ICIs in tumor therapy, irAEs are also widely reported. According to previous reports, about 70% of patients presented with irAEs after ICIs treatment, and 0.1–1% of them were cardiovascular toxicities (Brahmer et al., 2012; Topalian et al., 2012), and 5.0% of them were complicated by pneumonitis (Naidoo et al., 2017). The median onset time of myocarditis was about 30 days after the ICIs treatment, and 64% of the cases occurred after the first or second treatment (Moslehi et al., 2018; Salem et al., 2018). In this study, we observed fatal myocarditis combined with myasthenia gravis crisis, hepatic dysfunction, and delayed pneumonitis that occurred after 2 weeks of pembrolizumab initiation in a patient with lung LCNEC, and these symptoms have been successfully treated with implantation of cardiac pacemaker and high-dose steroids.

Although multiorgan failure occurred, fulminant myocarditis was likely the most critical cause of clinical deterioration in this patient. For patients with immune-related myocarditis, more common clinical presentations included acute onset heart failure, arrhythmias or atypical chest pain, and myositis (Mahmood et al., 2018). In this case, the patient was initially diagnosed with myocarditis accompanied by myositis according to imaging, biochemical markers, and clinical manifestations. Subsequent muscle biopsy results were consistent with our initial diagnosis. According to a previous study, the infiltrating T cell populations in myocardial and tumor tissues were similar (Johnson et al., 2016). Thus, we used muscle tissue to analyze the T cell infiltration with T cell receptor (TCR) repertoires. Compared with the tissue before myocarditis, the muscle tissue during the onset of myocarditis had specific T cell proliferation, which may be related to myocardial infraction (Supplementary Figure S2). Overlap index (OLI) was used to assess the similarity of TCR repertoires between tissue and peripheral blood sample and higher OLI also indicated the higher immune response (Hsu et al., 2016). An increase in OLI in the blood during the onset of myocarditis in our patient indicated that the simultaneous multiorgan failure was related to immune responses (Supplementary Figure S2).

There was no consensus for the treatment of immune-related myocarditis. Many reports confirmed that high-dose steroids should be used in patients with myocarditis (Brahmer et al., 2018, Frigeri et al., 2018, Heinzerling et al., 2016, Tay et al., 2017, Yang and Asnani, 2018). Cecilia et al. reported a 79-year-old patient with metastatic prostate cancer who developed myocarditis during treatment with nivolumab. Then, the patient was admitted and started on methylprednisolone 1 mg/kg/day and discharged on a prednisone taper after normalization of cardiac enzymes on day 4 (Monge et al., 2018). However, in a case reported by Johnson et al., the myocarditis in a metastatic melanoma patient due to nivolumab had been treated with a high dose of methylprednisolone but the patient was failed to rescue (Johnson et al., 2016). Because no immediate response of methylprednisolone in our patients, cardiac pacemaker implantation was performed immediately to restore the normal electrophysiological activity of the myocardium. As with the Johnson et al. study, high doses of steroids also did not have an immediate effect on our patients. The difference is that we had enough time to implant a temporary pacemaker to assist the electrophysiological activity of the heart muscle. Then, the patient was successfully treated with high doses of steroids as in the previous report. Therefore, if there is no immediate response to high-dose steroids, pacemaker implantation may be considered to help restore normal cardiac electrophysiological activity. Pneumonitis was one of the major irAEs of anti-PD-1/PD-L1 monoclonal antibodies. In our center, of the 150 patients treated with the pembrolizumab regimens, 2.7% (4/150) of the patients have developed various grades of immune-related myocarditis; however, severe (grades 3–4) myocarditis was rare; treatment was discontinued for 8.0% (12/150) of the patients with ≥ grade 2 pneumonia, of which 33.3% (4/12) had grade 3/4; and this patient was the first patient with fatal multiorgan failure to be noticed at our center. Almost all patients improved from immune-related myocarditis or pneumonia after steroid therapy, except for the patients in this study. Unexpectedly, delayed pneumonitis was found after myocarditis therapy in this patient. As reported, pembrolizumab-induced pneumonitis developed with variable intervals after treatment, ranging from 1 week to 2 years (Naidoo et al., 2017). However, delayed pneumonitis after myocarditis or other irAEs were not reported to the best of our knowledge. This case indicated that heterochronous irAE may occur even after multiorgan irAE.

Our study has several limitations. First, this was a single patient case report and more studies are needed to prove the feasibility of our study. Next, due to the clinical presentation of the irAE, several examinations were absent before treatment, including cardiac imaging and cardiac biopsies.

In conclusion, simultaneous multiorgan failure can develop in patients who underwent pembrolizumab therapy and high-dose steroids, and implantation of pacemakers was an effective therapy. Meanwhile, we should pay attention to the occurrence of heterochronous irAE in follow-up.

## Data Availability Statement

The original contributions presented in the study are included in the article/Supplementary Material; further inquiries can be directed to the corresponding author.

## Ethics Statement

The study involving human participants was reviewed and approved by the Ethics Committee of the First Affiliated Hospital of Guangzhou Medical University. The patient provided his written informed consent to participate in this study. Written informed consent was obtained from the individual for the publication of any potentially identifiable images or data included in this article.

## Author Contributions

CZ was the principal investigator for this study and was involved in project oversight; XX and FW played critical roles in data collection and auditing; MO, BL, YG, and SL facilitated the collection of clinical data; YQ, XL, ZX, ML, and DG performed statistical analyses; RC and CZ gave critical comments and suggestions and revised the article; all authors approved the final version of the manuscript.

## Funding

This work was supported by Wu Jieping Fund-Ministry of Health Project (Grant No.: 320.6750.18125); State Key Laboratory of Respiratory Disease-The Independent Project (Grant No.: SKLRD-QN-201720); State Key Laboratory of Respiratory Disease-The Open Project (Grant No.: SKLRD-OP-2018011); Guangdong High-Level University Clinical Cultivation Project (Grant No.: 2017-21020).

## Conflict of Interest

The authors declare that the research was conducted in the absence of any commercial or financial relationships that could be construed as a potential conflict of interest.
