# Supplementary figures and images for: Case Report: Fatal Multiorgan Failure and Heterochronous Pneumonitis Following Pembrolizumab Treatment in a Patient With Large-Cell Neuroendocrine Carcinoma of Lung
Source: Front Pharmacol. 2021 Jan 29;11:569466. doi: 10.3389/fphar.2020.569466 (PMC7878548; doi:10.3389/fphar.2020.569466)

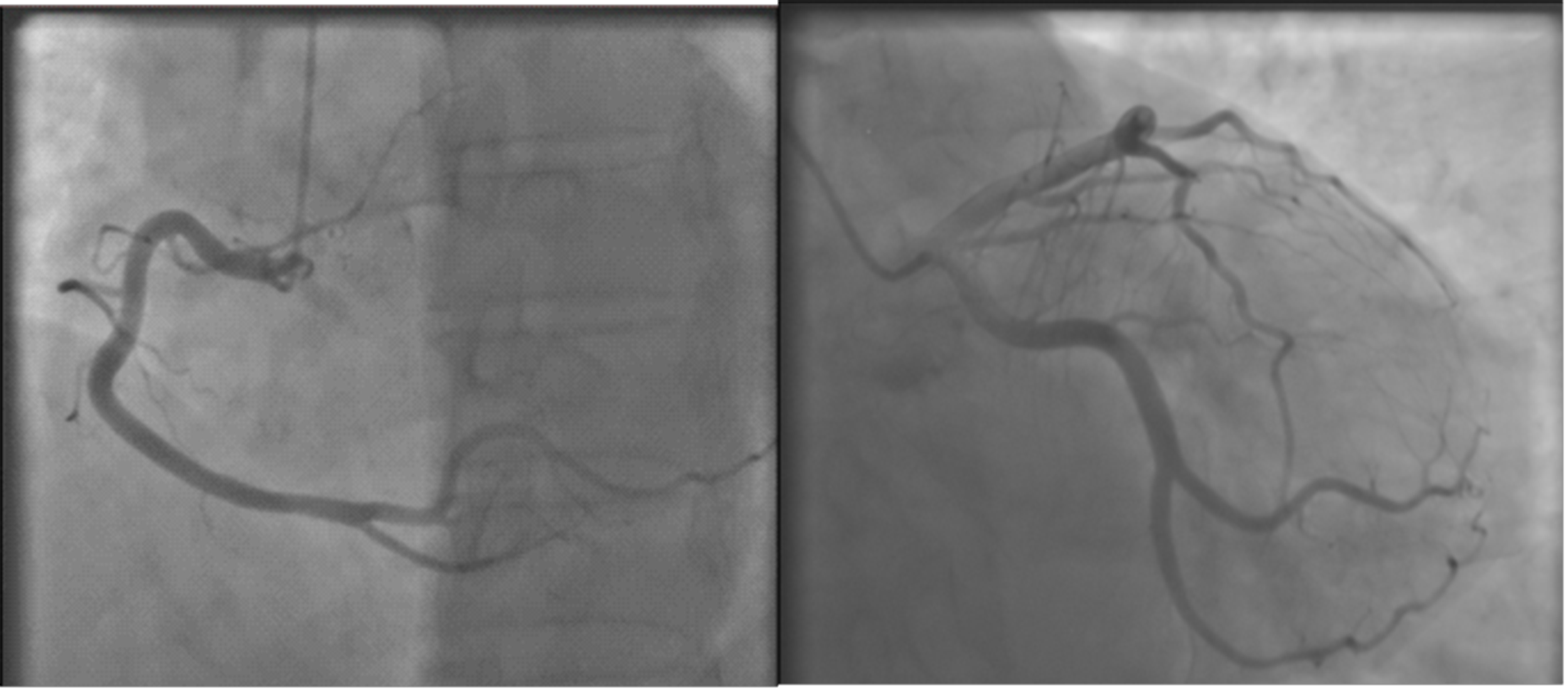

Supplement: Supplementary file 2 [file image1.tif]

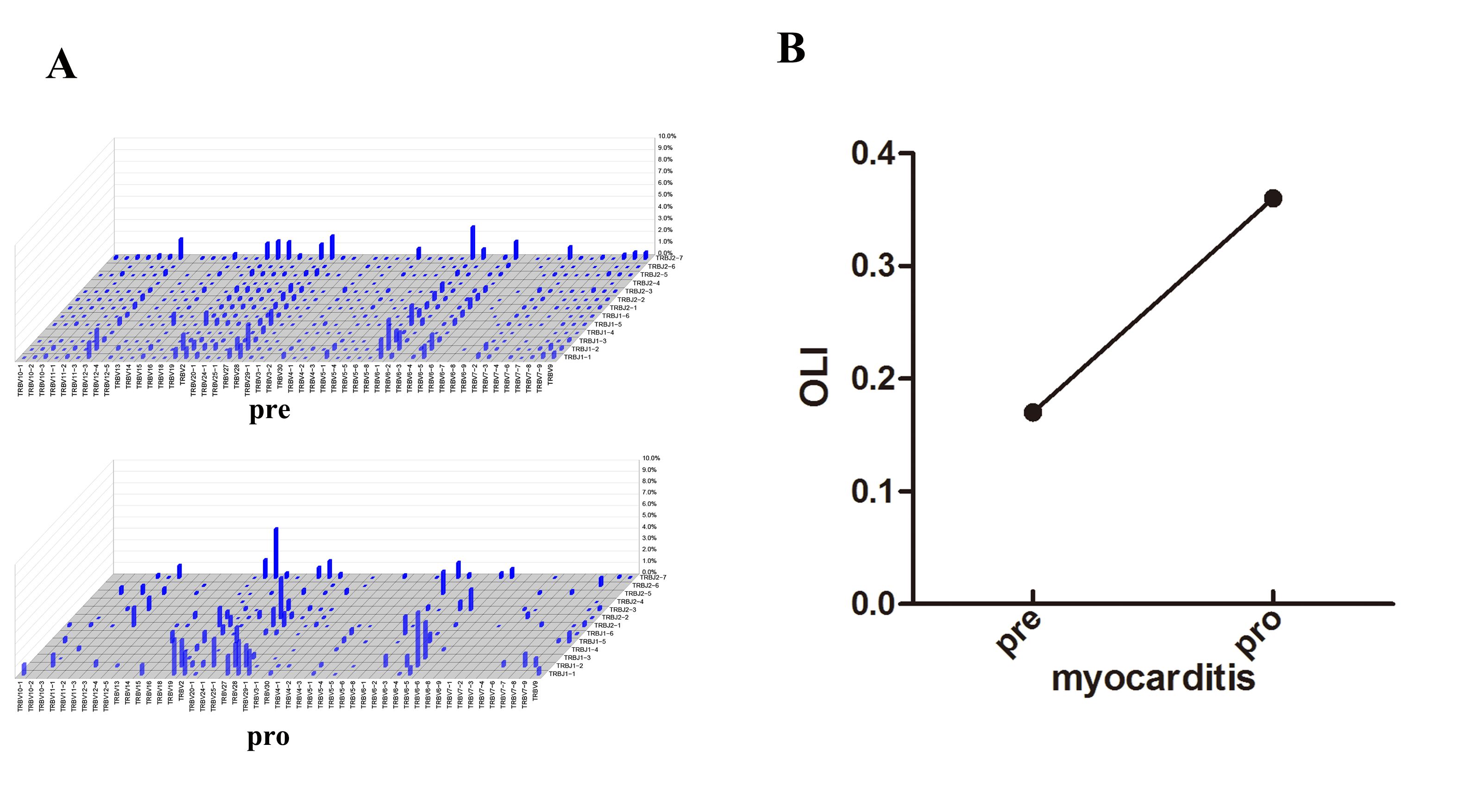

Supplement: Supplementary file 3 [file image2.tif]
